# The myogenic kinome: protein kinases critical to mammalian skeletal myogenesis

**DOI:** 10.1186/2044-5040-1-29

**Published:** 2011-09-08

**Authors:** James DR Knight, Rashmi Kothary

**Affiliations:** 1 Regenerative Medicine Program, Ottawa Hospital Research Institute, 501 Smyth Road, Ottawa, ON, K1H 8L6, Canada; 2 Department of Cellular and Molecular Medicine, University of Ottawa, 451 Smyth Road, Ottawa, ON, K1H 8M5, Canada; 3 Department of Medicine, University of Ottawa, 451 Smyth Road, Ottawa, ON, K1H 8M5, Canada

**Keywords:** protein kinase, satellite cell, myoblast, myocyte, myotube, proliferation, differentiation, fusion, hypertrophy, myogenesis

## Abstract

Myogenesis is a complex and tightly regulated process, the end result of which is the formation of a multinucleated myofibre with contractile capability. Typically, this process is described as being regulated by a coordinated transcriptional hierarchy. However, like any cellular process, myogenesis is also controlled by members of the protein kinase family, which transmit and execute signals initiated by promyogenic stimuli. In this review, we describe the various kinases involved in mammalian skeletal myogenesis: which step of myogenesis a particular kinase regulates, how it is activated (if known) and what its downstream effects are. We present a scheme of protein kinase activity, similar to that which exists for the myogenic transcription factors, to better clarify the complex signalling that underlies muscle development.

## Review

Embryonic myogenesis is a multistep process that begins with the commitment of an embryonic precursor to the myogenic lineage, followed by the proliferation of these committed myoblasts, the differentiation of myoblasts into postmitotic myocytes, and finally fusion of myocytes to form a multinucleated myotube. As the myotube matures, the syncytial cell becomes specialized for its particular function, with the bulk of the cytoplasm occupied by the contractile apparatus, and where the myotube/myofibre can further grow or hypertrophy in response to appropriate stimuli. Postnatal myogenesis is a similar process, except that fusion occurs primarily between myoblasts and preexisting myotubes, and where the role of the embryonic precursor is played by the quiescent satellite cell.

The process of myogenesis is controlled by several myogenic transcription factors that act as terminal effectors of signalling cascades and produce appropriate developmental stage-specific transcripts. The concerted roles that these transcription factors play is well known and well reviewed (see, for example, Sabourin and Rudnicki [1] and Le Grand and Rudnicki [2]). Paired-box protein 7 (Pax7) maintains a population of quiescent satellite cells and, together with myogenic factor 5 (Myf5), plays a role in the expansion of activated myoblasts. Myoblast determination protein (MyoD) is believed to determine the differentiation potential of an activated myoblast, and acts together with myogenin and myocyte enhancer factor 2 (MEF2) to drive differentiation. Finally, muscle-specific regulatory factor 4 (MRF4) is required for hypertrophy, although it may have other roles as well. Obviously, these transcription factors do not act alone, but exist as part of complex signalling cascades that control every stage of myogenesis. One of the major components of these cascades is the protein kinase, an enzyme that directs cell behaviour through the simple but reversible process of phosphorylation. Over 500 kinases exist in humans and mice [3,4]; however, a myogenic scheme of protein kinase activity, similar to that which exists for the above-named transcription factors, has not previously been elaborated.

**Figure 1:**
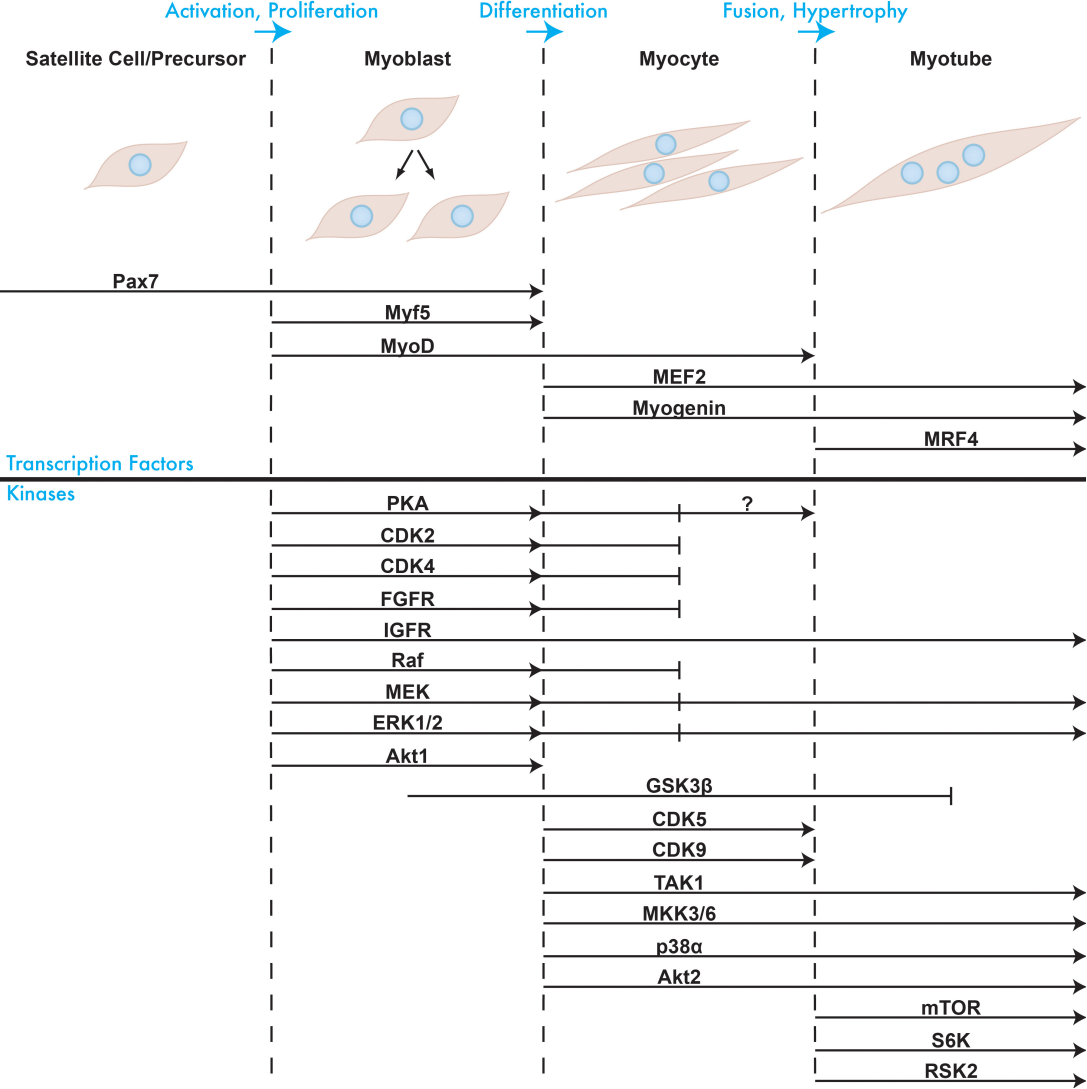
**Transcription factors and kinases regulating the different stages of myogenesis**. A graphical representation of myogenesis is shown. Embryonic precursors or quiescent satellite cells become activated to form proliferating myoblasts, which differentiate into myocytes that fuse to form a multinucleated myotube. The upper portion of the figure shows the myogenic transcription factors required for this process and the stages for which they are required. The lower portion shows the myogenic protein kinases and the stages that they regulate.

In this review, we summarize the involvement of the different protein kinases that participate in myogenesis. We discuss the stages they are required for, how they are activated during development/regeneration, and what the consequences of their activation are in terms of immediate phosphorylations and the downstream processes regulated. We discuss only developmental and regenerative skeletal myogenesis, in particular that of mammals, as cardiomyogenesis and the myogenesis of other vertebrate and invertebrate species contain unique features that require separate discussion. As dozens of kinases have been implicated in controlling some stage of myogenesis, this review covers the major players, those for which there is substantial evidence documenting their involvement (Figure 1).

### Protein kinase A

Protein kinase A (PKA), or cAMP-dependent protein kinase, was discovered over 40 years ago, the second protein kinase to be described [5]. It is involved in a multitude of cellular processes and has hundreds of substrates [6]. In its inactive form, it exists as a holoenzyme containing two catalytic and two regulatory subunits. Each regulatory subunit can bind cAMP, and binding triggers dissociation of the holoenzyme and the release/activation of the catalytic subunits. Elevation in intracellular cAMP levels is therefore the primary mechanism of activation for PKA.

**Figure 2:**
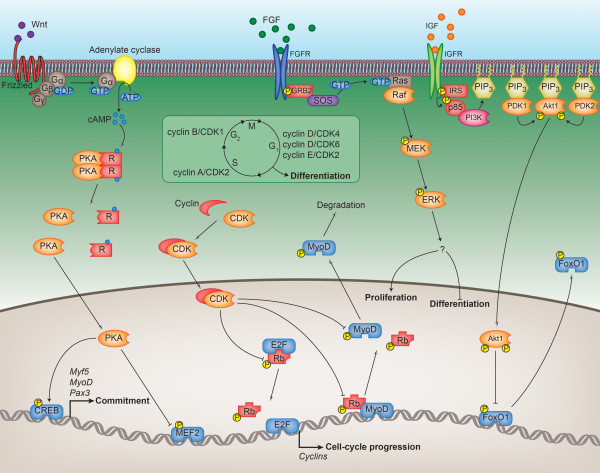
**Regulation of the early myogenic transcriptional program by the kinome**. The figure shows the mechanisms by which the kinases described in the text coordinate embryonic precursor activation, myoblast proliferation and the prevention of premature myoblast differentiation. Wnt1 and Wnt7a stimulation of precursor cells activates protein kinase A (PKA), which, through the phosphorylation of CREB, induces the expression of the myogenic transcription factors Myf5, MyoD and Pax3, resulting in the myogenic commitment of embryonic precursors. PKA then prevents the premature differentiation of proliferating myoblasts by phosphorylating and inhibiting the transcriptional activity of MEF2D. The cyclin-dependent kinases (CDKs) regulate cell cycle transitions and are activated at the appropriate time by the availability of their respective cyclins, depicted in the boxed inset. Cell cycle progression is achieved by the CDKs through the phosphorylation of Rb, which, when phosphorylated, is unable to bind and inhibit the E2F family of transcription factors that promote the expression of genes involved in cell division. Phosphorylation of Rb by the CDKs also prevents it from associating with and transactivating MyoD, thereby inhibiting cell cycle exit and differentiation. Cell cycle exit is further prevented by the proteolytic degradation of MyoD that results from direct CDK phosphorylation. The extracellular signal-regulated kinase (ERK) is activated by growth factors such as fibroblast growth factor and insulin-like growth factor (IGF), although the substrates ERK acts on to promote proliferation and inhibit differentiation are unknown. IGF also activates the Akt1 pathway and stimulates proliferation when myoblasts are subconfluent. Phosphorylation of FoxO1 by Akt1 prevents this transcription factor from accumulating in the nucleus, inhibiting the expression of genes involved in cell cycle exit, such as *p27*.

PKA is required at multiple stages during myogenesis, but an initial requirement is found during embryogenesis and the formation of myogenic precursors (i.e. myoblasts) within the dermomyotome (Figure 2). PKA activity is required for the expression of *Pax3*, *MyoD *and *Myf5 *in cells of the dermomyotome that will transition to form the myotome proper [7]. This activity is initiated by the release of Wnt1 and Wnt7a from the dorsal neural tube and surface ectoderm, respectively, which activate heteromeric G proteins and adenylate cyclase to produce cAMP, activating PKA. As PKA has many substrates, the expression of these myogenic genes is likely accomplished through several different targets, but at least one of these is the transcription factor cAMP response-element binding protein (CREB). Phosphorylation of CREB by PKA allows it to initiate the transcription of genes that contain a CRE element, two of which are *Pax3 *and *Myf5*. Chen *et al. *[7] showed that phosphorylated CREB is present at high levels in cells of the dermomyotome that express *Pax3*, *MyoD *and *Myf5 *and that this phosphorylation is critical for the induction of these genes. What is interesting about PKA with regards to myogenesis is that although it triggers the expression of several myogenic transcription factors, it also acts to suppress their transcription activity. It has been shown that PKA can inhibit the activity of Myf5, MyoD, myogenin and MEF2D without affecting their ability to bind DNA [8-10]. In the case of the MRFs, this appears to occur via an intermediary mechanism as opposed to direct phosphorylation, but in the case of MEF2D it is direct.

It is not clear what happens at the onset of differentiation with regards to PKA, although previous results suggest that PKA activity ultimately drops as differentiation proceeds, at least in C2C12 cells [11]. Obviously, its repressive effect on the MRFs and MEF2D must be removed for differentiation to occur, and this could arise through a reduction in cAMP levels, but what happens to cAMP upon differentiation is uncertain. Different groups have reported conflicting results regarding cAMP levels and their effect on myoblast differentiation in secondary cell lines [9,11-14]. What seems clear from a recent study utilizing primary myoblasts and C2C12 cells is that cAMP does not have an inhibitory effect on differentiation, but rather enhances both fusion- and differentiation-associated hypertrophy [15]. Although PKA activity would presumably be involved as a consequence of elevated cAMP, this was not convincingly shown, as the inhibitor used in that study (H89) is not absolutely specific to PKA. It was convincingly shown, however, that the appropriate localisation of PKA is critical for the positive myogenic effect of cAMP and that this appropriate localisation may be to lamellipodia. Early work on PKA and myoblast differentiation in L6 cells revealed that the establishment of appropriate levels of the regulatory and catalytic subunits of PKA is critical for differentiation [16,17]. It may be that PKA activity and cAMP are inhibitory to differentiation when present in certain areas (the nucleus, for example) and positive when found in other areas (lamellipodia). The repressive effect that PKA has upon the MRFs and MEF2D could be removed by a change in localisation or increased nuclear association of the catalytic subunit with its regulatory subunits, and this change may go hand in hand with a positive effect of PKA elsewhere in the cell. Ultimately, more detailed work on the role of PKA and cAMP during myoblast differentiation needs to be done to resolve these issues.

### Cyclin-dependent kinases

The cyclin-dependent kinases (CDKs) take their name from a catalytic dependence on the cyclin family of regulatory proteins. There are several cyclins and CDKs that collectively control cell cycle progression as well as other processes. The cyclins, and by extension the CDKs, can be divided into three major groups: the G_1 _cyclins, which regulate progression through G_1 _and entry into S phase; the mitotic cyclins, which regulate entry into mitosis; and the non-cell cycle cyclins, which have cell cycle-independent roles. This last group is not a typical classification, but we introduce it here because this group of cyclins/CDKs is important for myogenesis. The G_1 _cyclins include cyclins D and E, and are responsible for activating CDK4 (cyclin D), CDK6 (cyclin D) and CDK2 (cyclins D and E). The mitotic cyclins A and B activate CDK2 (cyclin A) and CDK1 (cyclin B). Levels of these cyclins are regulated by intrinsic cell cycle-derived signals, with the exception of cyclin D, which is regulated largely by extrinsic signals such as growth factors [18]. The final group of cyclins that have prominent roles outside the cell cycle include p35 and cyclin T, which activate CDK5 and CDK9, respectively. p35 is technically not a cyclin family member, but it activates CDK5 in the same allosteric manner as cyclins activate their CDKs and so we include it here.

In all cell types, the G_1 _and mitotic cyclins/CDKs regulate cell cycle progression and proliferation, and myoblasts are no different (Figure 2). One of the major mechanisms by which cell cycle progression is mediated is through CDK-dependent phosphorylation of the retinoblastoma protein (Rb). When phosphorylated, Rb is unable to bind and inhibit the E2F family of transcription factors, whose activities drive cell cycle progression. In proliferating myoblasts, the CDKs have an additional role in preventing precocious differentiation. Cyclin E/CDK2 and cyclin D/CDK4 can both block differentiation and the transcriptional activity of MyoD [19-24]. Cyclin E/CDK2 blocks MyoD-induced gene expression through the phosphorylation of Rb [22], preventing Rb from binding and transactivating MyoD [25], and triggering S phase entry instead of differentiation. Overexpression (or the natural accumulation in myoblasts) of MyoD is one well-known way to drive myogenic differentiation, even in nonmyogenic cell lines. Cyclin E/CDK2 can phosphorylate MyoD at serine 200 [26-28], which causes ubiquitination and degradation of this transcription factor during G_1 _[28,29], preventing its accumulation and a commitment to differentiation. Phosphorylation of MyoD at S200 is common to other CDKs, such as the mitotic cyclin B/CDK1 [26], which may prevent inappropriate MyoD accumulation during mitosis. In contrast to CDK2, cyclin D/CDK4 blocks MyoD activity through an as yet unclear mechanism that may involve direct binding [22,30,31]. Cyclin D/CDK4 can also block the activity of myogenin and all MEF2 isoforms [31]. Not much is known about how this occurs, but inhibition of MEF2C by CDK4 prevents the association of MEF2 with its transcriptional coactivator, glucocorticoid receptor-interacting protein 1 (GRIP1) [31]. Whether CDK6 also plays a role in preventing differentiation is unknown, although the mechanisms by which the CDKs block differentiation are likely much more complex than what we present here.

For myoblasts to differentiate, the cell cycle must be exited and the restraints the CDKs place on differentiation must be removed. Differentiation cues, such as serum withdrawal or cell-cell contact in cultured cells, elicit several effects that ultimately cause a decrease in G_1 _and mitotic CDK activity. The expression of cyclin D1, CDK1, CDK2 and CDK6 drop with differentiation, while cyclins A, B and E may also decrease [19-21,23,26,32-41]. At the same time as the expression levels of these proteins decline, there is an increase in the levels of the two families of CDK inhibitors (CKIs): the inhibitor of CDK4 family (INK4) and the cyclin-dependent kinase-interacting protein/kinase-inhibitory protein family (CIP/KIP). The INK4 family includes p15, p16, p18 and p19, and, despite the family name, these members also inhibit CDK6. The CIP/KIP members include p21, p27 and p57, and these members inhibit all G_1 _CDKs. There is substantial evidence demonstrating the importance of the CKIs for myoblast differentiation *in vitro *and *in vivo*. The expression levels of p16, p18, p19, p21, p27 and p57 all increase with differentiation [27,33,35,38,39,41-46], there is a sharp increase in p27 levels in the myotome at the onset of development [47], and mice lacking p21 and p57 form defective muscles [48]. When myoblasts are cued to differentiate, the CKIs bind and inhibit the G_1 _and mitotic CDKs [23,33,35,36,39,41], and do so throughout differentiation and even in adult tissue, which is important as not all cell cycle CDKs are lost with differentiation [20,23,27,35-38]. Unlike the other cyclins mentioned, cyclin D3 levels increase with differentiation, during which process this cyclin interacts strongly with CDK2 and CDK4 [19-21,36,37,39,41,49]. However, CDK-containing cyclin D3 complexes lack activity, suggesting that cyclin D3 may fulfil a necessary role as part of an inhibitory complex during differentiation. The end result of these changes in protein expression, whether it be cyclins, CDKs or CKIs, is a net loss of cell cycle CDK activity [26,32,33,35,37-39], hypophosphorylation of Rb [34,36,38], cell cycle exit, accumulation of MyoD [27], and leave for myoblasts to differentiate.

**Figure 3:**
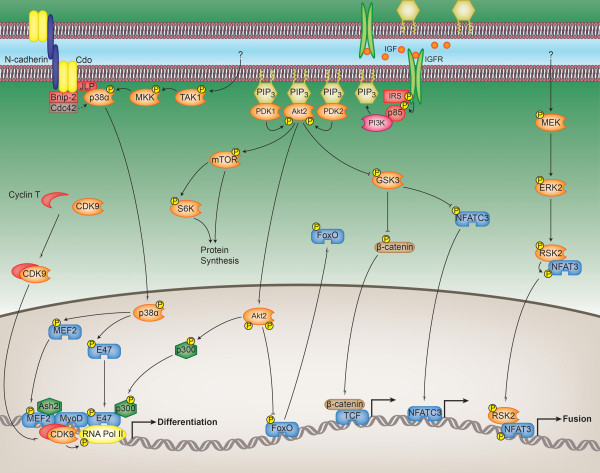
**Regulation of the late myogenic transcriptional program by the kinome**. The figure shows the mechanisms by which the kinases described in the text coordinate myoblast cell cycle exit, myoblast differentiation, myocyte fusion and myotube hypertrophy. Cell-cell contact and N-cadherin ligation, in conjunction with transforming growth factor-β-activated kinase 1 (TAK1) and MAP kinase kinase 3/6 (MKK), activate p38α. p38α induces cell cycle exit and differentiation through the phosphorylation of MEF2 and E47 that together with MyoD form part of an active myogenic transcriptional complex. A subunit of this complex is RNA polymerase II (RNA Pol II), which is phosphorylated and activated by cyclin-dependent kinase 9 (CDK9). Akt2, in response to IGF stimulation, phosphorylates the transcriptional coactivator and histone acetyltransferase p300, which is part of the same myogenic transcriptional complex. Activation of the Akt2 pathway promotes differentiation and hypertrophy by several other mechanisms as well. Akt2 phosphorylates and inactivates the FoxO family of transcription factors, whose activities are inhibitory to differentiation and hypertrophy. Phosphorylation of the mammalian target of rapamycin (mTOR) by Akt2 encourages protein synthesis/hypertrophy, partly through mTOR's phosphorylation and activation of the ribosomal protein S6 kinase 1 (S6K). Akt2 can also phosphorylate and inhibit glycogen synthase kinase 3β (GSK3). When active, GSK3 inhibits differentiation and hypertrophy through phosphorylation and cytoplasmic sequestration of NFATC3. Phosphorylation of β-catenin by GSK3 similarly prevents its nuclear accumulation and ability to activate the TCF/LEF family of transcription factors. Finally, activation of extracellular signal-regulated kinase 2 (ERK2) by an unknown stimulus promotes cell fusion through the phosphorylation and nuclear accumulation of NFAT3 via the 90-kDa ribosomal S6 kinase 2 (RSK2).

Once the cell cycle CDKs have been effectively silenced and the cell cycle exited, the non-cell cycle CDKs are important for promoting and establishing differentiation (Figure 3). These include CDK5 and CDK9, which are not inhibited by the CKIs discussed above. The expression of the CDK5-activating protein p35 is induced with myoblast differentiation and during muscle regeneration, the more stable and active calpain cleavage product of p35 (p25) increases as differentiation progresses, and the activity of CDK5 subsequently increases [50-53]. Dominant-negative CDK5 that lacks activity inhibits both differentiation and fusion [50,51,54], although the mechanisms by which CDK5 activity promotes these processes are not clear. In myoblasts, CDK5 can interact with, phosphorylate and regulate nestin [51], a negative regulator of differentiation, while nestin in turn can feed back and control CDK5 activity by preventing the processing of p35 into p25 [54]. Through a bidirectional relationship, CDK5 and nestin appear to control the rate at which myoblast differentiation occurs. Like CDK5, the activity of CDK9 also increases with differentiation, and this activity is critical for both *in vitro *differentiation and *in vivo *regeneration following injury [55-57]. Overexpression of it (or its activating cyclin T2) accelerates differentiation, at least in part through enhancing the activity of MyoD [55]. Cyclin T2/CDK9 can interact with and phosphorylate MyoD, although the consequences of phosphorylation are not known [55,57]. The interaction between these components, however, is critical for MyoD to induce gene expression. The recruitment of cyclin T2/CDK9 by MyoD to muscle-specific loci is believed to result in the phosphorylation and activation of RNA polymerase II by CDK9, thereby inducing transcription of myogenic genes [56]. Needless to say, what is known about these non-cell cycle CDKs and their role in differentiation is very partial, but highlights how the CDK family of kinases regulates myogenesis in a number of ways.

### Extracellular signal-regulated kinase (FGFR, Raf, MEK, RSK2)

Extracellular signal-regulated kinase (ERK) was first identified as an insulin-sensitive kinase that could phosphorylate the microtubule-associated protein 2, hence its original name 'MAP2 kinase' or 'MAP2K' [58]. It was later given the more general name 'ERK' [59], as its activity can be stimulated by a variety of growth factors/mitogens and it has many substrates in addition to MAP2. It is still generally known as 'MAPK', but with 'MAPK' now an acronym for 'mitogen-activated protein kinase'. There are several ERK isoforms, and there are other kinases that go by the name 'ERK', but generally, when used, the name refers to isoforms ERK1 and ERK2.

ERK1 and ERK2 (ERK1/2) belong to a well-defined pathway (Figure 2) that is activated by growth factor stimulation of a receptor tyrosine kinase, such as the binding of fibroblast growth factor (FGF) to its receptor (FGFR). Autophosphorylation of the growth factor receptor follows ligand binding and initiates the formation of an adaptor complex through Src homology 2 (SH2) domain-containing proteins such as GRB2. GRB2 interacts with the guanine-nucleotide exchange factor SOS, and localisation of these two proteins to the plasma membrane near the GTPase Ras allows SOS to catalyse GTP exchange and activation of Ras. GTP-bound Ras then binds and activates Raf, initiating the MAP kinase cascade. Raf is a MAP kinase kinase kinase (MAP3K) that phosphorylates and activates the dual-specificity MAP or ERK kinase (MEK), which in turn phosphorylates and activates ERK1/2.

Myoblasts/myocytes have a unique biphasic requirement for ERK activity. ERK1/2 is critical for growth factor-induced cellular proliferation, inhibitory to myoblast differentiation, and later required for myocyte fusion, or at least ERK2 appears critical to this last process. One previous publication also suggested that the Raf-MEK-ERK pathway might play a role in maintaining satellite cell quiescence [60], but further experimentation is required before this can be accepted. What is disappointing about the research that has been done on this pathway and its role during myogenesis, or rather on ERK's role in particular, is that virtually no ERK target phosphorylations have been studied or even identified. It is assumed that relevant substrates will be canonical ERK targets that have been studied in other cell types, but this has not been shown and there may very well be novel muscle-specific substrates as well. Research has instead focused on discovery and further description of the stages of myogenesis that ERK regulates, with some insight into secondary mechanisms, but almost nothing on direct substrates and their role in the myogenic process. With that in mind, we shall proceed with a discussion of what is known about the function of ERK and this pathway during myoblast proliferation and differentiation.

Evidence from primary cell cultures suggests a critical role for ERK in myoblast proliferation [61,62], which is supported by extensive data from secondary cell lines. In myoblasts, ERK activity can be stimulated by a variety of growth factors. Serum, a complex mixture of mitogens, activates ERK [63-66], but FGF [64,67-71], hepatocyte growth factor (HGF) [61], insulin-like growth factor (IGF) [66,67,70,72-74], leukaemia-inhibitory factor (LIF) [75], and platelet-derived growth factor (PDGF) [73,76] can do so in isolation. Not all of these growth factors elicit the same response from ERK, however. FGF, HGF and IGF activate ERK to induce or maintain proliferation [61,67,69-72,77], while PDGF does not but can enhance survival [73,76]. During proliferation, ERK activity prevents cell cycle exit during G_1 _[78], and FGF/ERK's role during myoblast proliferation may be to prevent cell cycle exit and promote entry into S phase [69,79]. How ERK accomplishes these functions, and particularly how different responses are elicited from it by different growth factors, is unknown. Of the different ERK-inducing growth factors, FGF has been the best studied in the context of myoblast proliferation, and the signalling cascade that results from FGF stimulation is as described above [69-71,80,81], although it should be mentioned that FGF appears to affect proliferation by an additional ERK-independent pathway as well [69,82].

Although almost nothing is known about how ERK positively affects myoblast proliferation, not much more is known about how it prevents premature differentiation, although it is clear that it does. ERK only mediates this effect for certain growth factors, however. IGF and FGF can both stimulate ERK activity, but once cells reach confluency in culture, IGF stimulation promotes differentiation [66,72] while FGF stimulation prevents it [64,68-70,76,83-89]. This is likely due to IGF's ability to induce other pathways in addition to that of ERK (see Akt section below), and demonstrates how the role that ERK activity is playing needs to be considered within the physiological context in which it occurs. In the context of FGF-induced activity, the Ras-Raf-MEK-ERK pathway is able to inhibit differentiation [62,64,70,78,81,88-100] by preventing the nuclear accumulation of MEF2 [96], and preventing the expression of certain myogenic factors, including MyoD [85-87,101-104], the CDK inhibitor p21 [94,95] and other transcriptional regulatory proteins [105]. ERK's and FGF's ability to prevent myoblast differentiation is supported by the biochemical observation that during differentiation FGF receptors are lost [106,107] and the activity of ERK decreases [52,66,81,92,95,97,108]. Again it appears that this critical role of ERK in blocking differentiation occurs specifically during G_1 _[84], possibly as an inhibitory cue that prevents the accumulation of proteins that would drive cells into a postmitotic phenotype. As mentioned, the substrates that ERK acts on to prevent myoblast differentiation are unknown.

ERK activity does initially decrease with myoblast differentiation, which is necessary for differentiating myoblasts to overcome the inhibitory effect that it has, but ERK's activity comes back on as differentiation proceeds [66,92,93,95,97,104,108-110]. ERK activity, and specifically that of the ERK2 isoform, is critical for myocyte fusion and survival (Figure 3) [77,92,93,95,104,109]. ERK can phosphorylate and activate the 90-kDa ribosomal S6 kinase 2 (RSK2), which positively regulates myocyte fusion through phosphorylation and transcriptional activation of nuclear factor of activated T cell 3 (NFAT3) [111]. ERK activity also stimulates the transcriptional activity of MyoD by an as yet to be described mechanism [104], and, contrary to ERK's role in myoblasts, it now enhances the expression of p21 [95,110]. There may be uncoupling of the Raf-MEK-ERK pathway during myocyte fusion as there are contradictory data on the function of Raf, with different reports describing both positive and negative roles for it [94,98,104,110], although it is clear that both MEK and ERK play positive roles. Similarly, FGF is certainly inhibitory to fusion, and so the growth factor or mechanism stimulating ERK activity in myocytes is unknown and the pathway promoting this activity needs further elucidation.

### p38α (TAK1, MKK3/6)

The p38 family of MAPKs are closely related to the ERK MAPKs discussed above, and take their rather unimaginative name from their apparent molecular weight. The α isoform of the p38 family was initially identified as an effector of the cellular stress response [112-115], but has also been shown to be critical for the differentiation of numerous cell types [116-121]. There are three other p38 isoforms, β, γ and δ, but only p38α appears uniformly critical for differentiation, with the other isoforms either unnecessary or with insufficient evidence supporting an essential role.

At the turn of the century, several groups reported a critical role for one of or both the p38α and β isoforms during myoblast differentiation [95,97,122-125]. It was found that p38 activity was induced during differentiation in culture and that inhibition of the α and β isoforms blocked the induction and/or activation of myogenic and muscle-specific genes, as well as prevented myocyte fusion. These studies were all performed with secondary cell lines (C2C12, L6E9, L8 and transformed 10T1/2), but *in vivo *work has confirmed that p38 activity is indeed critical for myoblast differentiation. During embryonic development, p38 activity is induced in somites, and inhibition does not affect the myogenic commitment of cells but does block the induction of the myotomal muscle marker myosin light chain 3F (MLC3F) [126]. Most recently, the group of Pura Muñoz-Cánoves has demonstrated through isoform-specific knockout in mice that p38α is absolutely critical for the differentiation of primary myoblasts, while β and δ are not necessary for either differentiation or cardiotoxin-induced regeneration, and the γ isoform appears necessary only for optimal fusion of myoblasts [127-129]. It should be noted, however, that a discrepancy may exist between primary myoblasts and C2C12 cells, as the α, β and γ isoforms all appear to be essential for C2C12 differentiation [125,130,131], highlighting that the model system being used always needs to be taken into consideration.

Cell-cell contact in myoblast cultures triggers precocious differentiation, and contact is at least one mechanism by which p38α is activated (Figure 3). N-cadherin ligation between cells initiates the formation of a complex that includes the cell surface protein Cdo and scaffolding proteins that recruit p38 in addition to other components [132-134]. Precisely how this complex results in p38 activation is not known, but complex recruitment of the GTPase Cdc42 is required for p38 phosphorylation. However, as noted by Kang *et al. *[133], although Cdo complex formation appears to be a major mechanism behind p38 activation in differentiating myoblasts, it is likely not the only mechanism, as there are additional ways to activate p38 in the absence of Cdo complex components. Transforming growth factor β-activated kinase 1 (TAK1) is an upstream activating MAP3K that is essential for myoblast differentiation in a p38-dependent manner [135], and activation of this kinase is traditionally associated with transforming growth factor (TGF) stimulation as opposed to N-cadherin ligation. TAK1 can phosphorylate and activate MAP kinase kinase 3/6 (MKK3/6), and numerous studies have demonstrated a requirement for MKK3/6 activity in the initiation of myoblast differentiation, again in a p38-dependent manner. Whether N-cadherin ligation and Cdo are coupled to TAK1 and MKK3/6 is not known, and so it is not possible to present a clear pathway for p38 activation during differentiation.

Once activated, p38 is involved in multiple prodifferentiation processes (Figure 3). It has a powerful ability to trigger cell cycle exit, and can even force cell cycle exit in rhabdomyosarcoma cells [136]. The mechanisms by which it does so have not been well elucidated, but it can downregulate canonical proliferation markers such as cyclins A, D and E, as well as phosphorylated Rb [127,128,137]. Chromatin remodelling is a candidate mechanism by which p38 activity might trigger the downregulation of cell cycle-related genes. p38 can phosphorylate the histone-lysine N-methyltransferase EZH2, the catalytic subunit of the polycomb repressive complex 2 (PRC2), with phosphorylation of EZH2 necessary for PRC2's association with the transcriptional repressor YY1 and subsequent chromatin remodelling [138]. One target of this complex in myoblasts is the *Pax7 *promoter, and downregulation of Pax7 is a necessary step before differentiation can occur.

At the same time as p38 creates a repressive chromatin environment for *Pax7 *and possibly other genes, it creates a permissive environment at myogenic loci. p38 phosphorylates the BAF60 subunit of the SWI-SNF chromatin remodelling complex, and p38 recruits this complex to differentiation-specific loci [137,139]. Through phosphorylation of MEF2D, p38 recruits an Ash2l-containing complex to myogenic loci during differentiation, which results in the marking of these genes for expression [140]. As a permissive environment is created at these loci, p38 further stimulates gene expression through the phosphorylation of additional myogenic transcription factors, including MEF2C [95,123,126] and E47 [141]. Phosphorylation of MEF2C is necessary for its transcriptional activation, and E47 phosphorylation allows heterodimerisation with and activation of MyoD. p38 also plays a critical role in activating other myogenic factors. Nuclear translocation of p65 during differentiation is p38-dependent [142], as is MyoD activity [95,123,125,127,136], partly through E47 phosphorylation and heterodimerisation but likely via other means as well. Ultimately, through these and possibly other mechanisms, p38 has the ability to affect the expression of a multitude of genes. Some of those directly relevant to differentiation and not already mentioned include Akt [143,144], caveolin 3 [124] and IGF2 [145].

The responsibility of p38 during myoblast differentiation is not limited to gene regulation, but includes a critical role in other processes as well. Briata *et al. *[146] showed that p38 phosphorylates the mRNA decay-promoting KH type-splicing regulatory protein (KSRP). Phosphorylation prevents KSRP from associating with select transcripts, resulting in transcript stabilization, and in the case of differentiating myoblasts this allows for the accumulation of mRNA for at least two very critical myogenic proteins: the CDK inhibitor p21 and myogenin. Also, current work from our own laboratory shows that during myoblast differentiation active p38 accumulates in the cytoplasm and can phosphorylate dozens of cytosolic proteins with a variety of known functions, suggesting that the role of p38 during myogenesis likely goes far beyond gene regulation (JDR Knight, R Tian, REC Lee, F Wang, H Zou, LA Megeney, D Figeys, R Kothary, unpublished work).

Finally, it needs to be mentioned that the literature is not in complete consensus regarding the role of p38 during myoblast differentiation. A potentially conflicting result was published by Weston *et al. *[147], who showed that inhibiting p38α/β in a mixed culture of primary limb mesenchymal cells supports and accelerates the terminal differentiation of myocytes. Specifically, cells that already express myosin heavy chain appear to undergo accelerated fusion and/or hypertrophy, along with an increased expression of myogenic markers following p38 inhibition. These results suggest that in this type of heterogeneous environment, p38 activity, in concert with a particular milieu of factors released by nonmyogenic cells, may serve to restrict the late stages of myocyte differentiation, or that obstructing p38 activity in nonmyogenic cells present in the coculture results in the release of potent myogenic factors that drive terminal myocyte differentiation even in the absence of active p38. As no further work has been done on this model system and more experiments are required, it is not possible to reconcile these findings with the extensive data produced using other models.

### Akt (IGFR, GSK3β, mTOR, S6K)

The protein kinase Akt first became known as the product of the oncogenic *v-akt *gene of the Akt8 murine retrovirus [148]. The retroviral oncogene has three mammalian cellular homologues (*Akt1*, *Akt2 *and *Akt3*) that code for a protein kinase with an N-terminal pleckstrin homology (PH) domain [148-153]. Owing to its independent discovery by three separate groups, it has two additional names: protein kinase B (PKB) and the related to the A and C kinases (RAC-PK), on the basis of its homology [149,151].

Akt forms part of a well-studied pathway (Figure 3), and for a review, see, for example, the articles by Glass [154] and Franke [155]. This pathway mediates the effects of insulin and IGF and includes several kinases that shall be discussed together here, although focus is placed on Akt. The pathway is activated by the binding of IGF to the IGF receptor (or of insulin to the insulin receptor), and, like most growth factor receptors, IGFR contains a tyrosine kinase domain that autoactivates upon ligand binding. A principal target of IGFR is the insulin receptor substrate (IRS), which, when phosphorylated, recruits the lipid kinase phosphatidylinositol 3-kinase (PI3K) through the SH2 domain of its regulatory subunit (p85), triggering activation of the catalytic subunit. PI3K produces the membrane-bound phosphatidylinositol (3,4)-bisphosphate (PI(3,4)P_2_) and phosphatidylinositol (3,4,5)-trisphosphate (PI(3,4,5)P_3_) from PI(4)P and PI(4,5)P_2_, respectively. These phosphoinositide products localise PH domain-containing proteins to the plasma membrane, including Akt, the constitutively active phosphoinositide-dependent kinase 1 (PDK1), and "PDK2". The colocalisation of these kinases allows for PDK1 and PDK2 to phosphorylate Akt at distinct sites, with both phosphorylations necessary for activation. PDK2 is not a single kinase but rather a group of kinases [156], any one of which has the ability to phosphorylate Akt at the required site. Two major kinase targets of Akt are the mammalian target of rapamycin (mTOR) and glycogen synthase kinase 3β (GSK3β). Phosphorylation of mTOR by Akt activates it, resulting in an increase in protein synthesis, while Akt's phosphorylation of GSK3β inactivates this kinase, thereby removing the restraint that GSK3β places on differentiation and hypertrophy. One final, well-characterized member of this pathway is the ribosomal protein S6 kinase 1 (S6K), which is phosphorylated and activated by mTOR to positively and further regulate protein translation.

This pathway, with Akt at its heart, is activated by IGF or insulin stimulation, but there is evidence to suggest that Akt can be activated by other mechanisms in muscle cell lines. Elia *et al. *[157] showed that Sonic hedgehog (SHH) can stimulate Akt phosphorylation and myogenic gene expression, and, similar to work done on the p38 pathway, Bae *et al. *[158] showed that Akt can be activated from cell-cell contact through Cdo activation and the recruitment of the Akt-interacting partner APPL1. There is evidence to suggest that APPL1 may function downstream of insulin in myoblasts [159,160], indicating that cell-cell contact and insulin/IGF may cooperate to activate Akt. Whether SHH also cooperates with this pathway or stimulates one in parallel is unclear, but there is certainly more to be discovered about the mechanisms of Akt activation.

While the pathway of Akt activation requires additional elaboration, the importance of the IGF-Akt axis to myogenesis cannot be debated. It has been demonstrated in culture that IGF is critical to, and a potent stimulator of, myoblast differentiation and hypertrophy, and that muscle cell lines upregulate IGF2 upon differentiation [87,161-167]. These results carry over *in vivo*, as IGF overexpression in mice triggers myoblast differentiation, myofibre hypertrophy and regeneration [168-170]. Several studies have shown that Akt activity is induced during myoblast differentiation, and that its activity is critical for the induction of differentiation and hypertrophy both in culture and *in vivo *[52,94,125,144,171-175]. IGF can also have a positive effect on myoblast proliferation under certain conditions, and Akt may be critical for proliferation as well [176,177], although the details regarding this pathway are poorly understood. We shall discuss the proliferative capabilities of IGF and Akt in greater detail below after first introducing the different Akt isoforms and their respective myogenic responsibilities.

IGF can activate any of the three Akt isoforms, and currently both Akt1 and Akt2 have been implicated in myogenesis, while Akt3 has not. There is very strong evidence to suggest that isoforms 1 and 2 are required at different stages, although how their activation is differentially controlled is unknown. Protein levels of Akt1 remain constant from proliferating to differentiating cells, whereas the levels and activity of Akt2 increase with differentiation [143,176,178-181]. Consistent with these observations, Akt2 drives differentiation [176,177,181-183], while Akt1 appears critical to myoblasts for proliferation but is dispensable for differentiation and may even be inhibitory to the latter process when activated alone [176,177,183,184]. Conversely, Akt2 is dispensable for proliferation and cannot rescue Akt1 knockdown in proliferating myoblasts [176,177]. It should be noted, however, that overexpression of a constitutively active mutant of either isoform can initiate and drive differentiation, but this is likely an artefact that results from artificially elevated Akt levels. It is difficult to be conclusive at the moment, especially as little work has been done *in vivo *or in primary cells, but there is certainly strong evidence to support distinct roles for Akt1 and Akt2 during myogenesis.

When myoblasts are initially treated with IGF, there is a proliferative response and differentiation is prevented [67,72,74,177,185-187]. This response is induced largely when myoblasts are subconfluent and is mediated in part by IGF-induced phosphorylation of ERK1/2, as well as Akt1 (Figure 2). Few targets of Akt1 in proliferative myoblasts are known, but once activated, Akt1 phosphorylates the cyclin kinase inhibitor p21, triggering its dissociation from CDK2 and leading to cell cycle progression [74,177]. Akt can also phosphorylate forkhead box protein O1 (FoxO1) in myoblasts, with phosphorylation blocking nuclear translocation of the transcription factor and inhibiting expression of FoxO1-regulated transcripts such as the CDK inhibitor *p27 *[187]. Evidence suggests that this IGF proliferative pathway can be turned off either by inhibiting ERK1/2, or through the activation of Akt2 [72,94,99,177]. Once confluent, cell-cell contact is known to antagonize ERK1/2 activation in other cell types [188-191], and in myoblasts confluency induces p38 activity as described in the previous section, which in turn leads to the upregulation of *Akt2 *transcript levels [143]. Contrary to Akt1, Akt2 interacts with p21 but does not phosphorylate it, and instead appears to prevent phosphorylation by Akt1 [177]. This Akt2-p21 complex can then inhibit CDK2 and allow cell cycle exit and differentiation. Hence the switch from an IGF-induced proliferative response to an induction of differentiation may be controlled largely by the degree of cell-cell contact present.

Once Akt2 becomes activated, it triggers myoblast cell cycle exit (Figure 3). It does so by phosphorylating the pituitary homeobox 2 (Pitx2) transcription factor (which cannot be phosphorylated by Akt1) [192]. Pitx2 interacts with the mRNA binding protein HuR to stabilize *cyclin D1 *transcript levels to maintain proliferation, while Akt2 phosphorylation of Pitx2 causes dissociation of this complex and degradation of *cyclin D1 *mRNA. Once cell cycle exit has occurred, Akt's phosphorylation (and inhibition) of the FoxO family of transcription factors now allows differentiation to occur [193], as these transcription factors, although apparently necessary for cell cycle exit in myoblasts [187], are inhibitory to differentiation. Akt activity is also critical for the production of myogenic transcripts, partly through positive regulation of MyoD and MEF2C transcriptional activities [172,181,194]. Akt can phosphorylate the transcriptional coactivator p300, which results in the formation of an active p300-MyoD complex [137]. In myoblasts, MyoD and MEF2 activities are suppressed, partly from being bound to the transcriptional repressor prohibitin 2 (PHB2). Akt2 can remove this repression through binding to and downregulating PHB2 [183,195], allowing MyoD and MEF2 transcriptional activation, although whether Akt2-mediated phosphorylation of PHB2 occurs is unknown. A further factor that triggers differentiation in response to Akt activity is the phosphorylation and inactivation of GSK3β. When active, GSK3β represses myoblast differentiation and fusion [182,196-198] through inhibitory phosphorylations of β-catenin and NFATC3. The silencing of GSK3β activity by Akt allows for the accumulation and nuclear translocation of β-catenin [182,198], resulting in the activation of the TCF/LEF family of transcription factors. GSK3β's phosphorylation of NFATC3 hides this transcription factor's nuclear localisation signal, thereby preventing nuclear accumulation and transcription of its dependent genes, while Akt's phosphorylation and inhibition of GSK3β allows this NFATC3 accumulation to occur [197,198]. The end result of GSK3β inactivation is the activation of transcription factors that initiate the production of numerous myogenic transcripts.

Following commitment to differentiation, Akt is further required for the growth/hypertrophy of myotubes (Figure 3). Its multifaceted role during hypertrophy is emphasized by the fact that the exogenous overexpression of myogenic factors such as MyoD or myogenin cannot compensate for the absence of Akt activity during this process [199]. During hypertrophy, Akt is still responsible for phosphorylating and inactivating GSK3β as it was at the onset of differentiation, as GSK3β is inhibitory to both stages of myogenesis [173,198,200,201]. Similarly, Akt's phosphorylation of the FoxO family of transcription factors is necessary not just for differentiation but also for hypertrophy. FoxO activity stimulates expression of the atrophy-inducing, muscle-specific ubiquitin ligases MAFbx and MuRF1 [202,203], and Akt therefore blocks the expression of these ligases. One of the most well-studied downstream targets of Akt in muscle is mTOR, whose phosphorylation and activity are induced during hypertrophy [204]. mTOR itself is in fact required for both differentiation and hypertrophy [72,122,174], but its kinase activity is required only for the latter [204-209]. Approximately 90% of the genes regulated by IGF in differentiating myoblasts are mTOR-dependent [210], emphasizing the importance of this kinase as a hypertrophic IGF effector. mTOR, together with S6K, an mTOR substrate whose activity is induced during and critical to hypertrophy [72,108,122,175,204,205,209,211], trigger protein synthesis and growth by initiating cap-dependent translation. mTOR activity also leads to the upregulation of miR-1, which inhibits HDAC4 expression, thereby allowing the upregulation of critical myogenic genes, including the profusion protein follistatin [212].

A substantial amount of research on the IGF-Akt signalling axis has been conducted, and we have briefly summarized it here. Further research regarding the distinct roles of Akt1 and Akt2 is required: if, in fact, there is a distinction; how they are differentially regulated; and the similarities and differences between downstream targets. Over 100 Akt substrates are known, but very few have been studied during myogenesis. We have described the handful of substrates that have been studied, but this must be a very incomplete picture, and so there is still much room for further exploration.

## Conclusions

As we have reviewed here, the differential activation (and inhibition) of distinct protein kinases acts to control the formation of a mature myotube from a population of embryonic precursors or satellite cells. Although there is more to be discovered, a synthesis of the available information reveals a kinase hierarchy that coordinates myogenesis in a fashion analogous to the myogenic transcription factors. Initially, during development, and likely via analogous mechanisms during juvenile and adult myogenesis, the presence of specific Wnts induces PKA activity and the myogenic commitment of precursors to form a pool of dividing myoblasts. Growth factors, and likely other extrinsic components, then activate ERK1/2, Akt1 and cyclin D/CDK2, 4 and 6 to promote proliferation, which, along with PKA, act simultaneously to restrict differentiation. Intrinsic cell cycle-derived signals regulate the levels of cyclins A, B and E, which, together with their respective CDKs, promote cell cycle progression and inhibit differentiation. As the myoblast population expands to the threshold of available space, cell-cell contact can turn off ERK, and a decline in certain growth factors can further silence ERK and downregulate cyclin D/CDK2, 4 and 6 activity, to inhibit additional proliferation. With the silencing of ERK and cell cycle CDK activity, and proliferation lessening, cell-cell contact can promote differentiation through p38 and possibly Akt2 as well. Certain unknown cues may also relocalise PKA activity to remove its restraint on differentiation while allowing it to have a positive effect elsewhere in the cell. Similarly, undefined signals lead to an upregulation of p35 and cyclin T levels to induce the activity of CDK5 and 9, respectively. An increasing concentration of IGF, which is released during differentiation, stimulates Akt2 activity to drive differentiation and hypertrophy, and elevated IGF levels may also induce ERK2 activity at later time points to promote fusion. Finally, as differentiation progresses, p38 activity relocalises to the cytoplasm, where it promotes the mid to late stages of differentiation.

The protein kinase scheme we present herein demonstrates how a number of kinases are used by myogenic cells to transition from state to state, and the intricate signalling used to coordinate the proper development of muscle tissue. As complex as is the picture we offer, it is only partial. Not only does more need to be known about the kinases we have discussed, but there are many other kinases that have been implicated in controlling some aspect of myogenesis (Table 1). As research on muscle development continues, it will be interesting to learn how these other kinases fit in with the picture presented here. It is important to state that much of what is known about kinases and myogenesis relates either to myoblast proliferation, differentiation or myotube hypertrophy, while very little is known about the kinase signalling that regulates satellite cell quiescence and activation, as well as myocyte fusion. While Pax7 is a well-established marker and regulator of satellite cells, almost nothing is known about the protein kinases that regulate satellite cell quiescence or maintenance. With respect to fusion, not only is there limited information about the kinases that control this process, but little is known about it in general, which is surprising, considering its obvious importance to the most unique feature of muscle tissue: the multinucleated cell. Although fusion and hypertrophy are the end stage of our description of myogenesis, further stages could be added, including precursor migration, sarcomere formation and neuromuscular junction development. The prospect of a comprehensive review on myogenic signalling is an almost overwhelming one, due to the number of different cell states, processes and network complexity involved, but we hope to have begun clarifying it.

**Table 1:** Other protein kinases implicated in myogenesis

Stage^a^	Kinase	Full name	Mechanism^b^	References
Satellite cell activation	c-Met^c^	*N*-methyl-*N'*-nitro-*N*-nitrosoguanidine (MNNG) human osteosarcoma (HOS) transforming oncogene cellular homologue	?	[213-215]
Proliferation and/or inhibit differentiation	JAK1	Janus/just another kinase 1	Phosphorylation and activation of STAT1	[216]
	p38γ	p38γ mitogen-activated protein kinase	Phosphorylation and repression of MyoD activity	[217]
	ROCK1	Rho-associated kinase 1	Blocks activation of the Akt differentiation pathway	[218-220]
Differentiation	c-Abl	Abelson murine leukaemia viral oncogene cellular homologue	Activation of p38	[221,222]
	CaMK1/4	Calcium/calmodulin-dependent protein kinases 1 and 4	Phosphorylates HDAC5, thereby releasing repression of MEF2	[223-226]
	DYRK1B	Dual-specificity tyrosine phosphorylation-regulated kinase 1B	Phosphorylates HDAC5 and HDAC9, thereby releasing repression of MEF2	[227,228]
	MAPK7^d^	Mitogen-activated protein kinase 7	Phosphorylation and activation of MEF2C	[229,230]
	JAK2	Janus/just another kinase 2	Phosphorylation and activation of STAT2 and STAT3	[216,231]
	PKCζ	Protein kinase Cζ	Activation of CDK5	[53]
	PKD2	Protein kinase D2	Activation of MEF2D and repression of Pax3	[232]
Fusion	cGK1	Cyclic GMP-dependent protein kinase 1	Phosphorylation and inactivation of FoxO1	[233]
	FAK	Focal adhesion kinase	Fusogen expression	[234,235]
	PKCθ	Protein kinase Cθ	Activation of FAK	[234]
Hypertrophy	ROCK2	Rho-associated kinase 2	Activation of ERK2 and S6K	[236]

The protein kinases listed are those that have been clearly implicated in regulating some aspect of myogenesis but have not been studied in detail.

^a^The stage of myogenesis regulated by the indicated kinase. ^b^The mechanism the indicated kinase has been implicated in regulating. ^c^Also known as hepatocyte growth factor/scatter factor receptor (HGFR/SFR). ^d^Also known as big MAP kinase 1 (BMK1) or extracellular signal-regulated kinase 5 (ERK5).

## Abbreviations

Akt: v-akt murine thymoma viral oncogene cellular homologue, a.k.a. protein kinase B (PKB), a.k.a. the related to the A and C kinases (RAC-PK); APPL1: adapter protein containing PH domain, PTB domain and leucine zipper motif 1; Ash2l: Ash2-like methyltransferase; BAF60: BRG1-associated factor 60; Cdc42: cell division control protein 42; CDK: cyclin-dependent kinase; Cdo: cell adhesion molecule-related/downregulated by oncogenes; CIP/KIP: cyclin-dependent kinase-interacting protein/kinase-inhibitory protein; CKI: CDK inhibitor; CREB: cAMP response element-binding protein; ERK: extracellular signal-regulated kinase, a.k.a. microtubule-associated protein 2 kinase (MAP2K); EZH2: enhancer of zeste homologue 2; FGF: fibroblast growth factor; FGFR: fibroblast growth factor receptor; FoxO1: forkhead box protein O1; GFR: growth factor receptor; GRB2: growth factor receptor-bound protein 2; GRIP-1: glucocorticoid receptor-interacting protein 1; GSK3β: glycogen synthase kinase 3β; HDAC: histone deacetylase; HGF: hepatocyte growth factor; HuR: human antigen R; IGF: insulin-like growth factor; IGFR: insulin-like growth factor receptor; INK4: inhibitor of cyclin-dependent kinase 4; IRS: insulin-receptor substrate; KSRP: KH type-splicing regulatory protein; LIF: leukaemia-inhibitory factor; MAFbx: muscle atrophy F box; MAPK: mitogen-activated protein kinase; MAP3K: mitogen-activated protein kinase kinase kinase; MEF2: myocyte enhancer factor 2; MEK: dual-specificity MAP or ERK kinase; MKK3/6: mitogen-activate protein kinase kinase 3/6; MRF4: muscle-specific regulatory factor 4; mTOR: mammalian target of rapamycin; MuRF1: muscle RING finger 1; Myf5: myogenic factor 5; MyoD: myoblast determination protein; NFAT3: nuclear factor of activated T cell 3; p38: p38 mitogen-activated protein kinase; p65/RelA: v-rel reticuloendotheliosis viral oncogene cellular homologue A; Pax7: paired-box protein 7; PDGF: platelet-derived growth factor; PDK1: phosphoinositide-dependent kinase; PH: pleckstrin homology; PHB2: prohibitin 2; PI3K: phosphatidylinositol 3-kinase; PI(3,4)P_2_: phosphatidylinositol (3,4)-bisphosphate; PI(3,4,5)P_3_: phosphatidylinositol (3,4,5)-trisphosphate; PI(4)P: phosphatidylinositol 4-phosphate; PI(4,5)P_2_: phosphatidylinositol (4,5)-bisphosphate; Pitx2: pituitary homeobox 2; PKA: protein kinase A, a.k.a. cAMP-dependent protein kinase; PRC2: polycomb repressive complex 2; Rb: retinoblastoma protein; RSK2: 90-kDa ribosomal S6 kinase 2; S6K: ribosomal protein S6 kinase 1; SH2: Src homology 2; SHH: Sonic hedgehog; SOS: son of sevenless; STAT: signal transducer and activator of transcription; SWI-SNF: switch/sucrose nonfermentable; TAK1: transforming growth factor β-activated kinase 1; TCF/LEF: T-cell factor/lymphoid enhancer-binding factor; TGF: transforming growth factor; Wnt: wingless/int; YY1: yin and yang 1.

## Competing interests

The authors declare that they have no competing interests.

## Authors' contributions

JDRK wrote the manuscript. RK revised and edited the manuscript. Both authors read and approved the final manuscript.
